# Construction and Characterization of an *Aeromonas hydrophila* Multi-Gene Deletion Strain and Evaluation of Its Potential as a Live-Attenuated Vaccine in Grass Carp

**DOI:** 10.3390/vaccines9050451

**Published:** 2021-05-03

**Authors:** Jihong Li, Shilin Ma, Zhi Li, Wei Yu, Peng Zhou, Xiang Ye, Md. Sharifull Islam, Yong-An Zhang, Yang Zhou, Jinquan Li

**Affiliations:** 1 State Key Laboratory of Agricultural Microbiology, College of Fisheries, Huazhong Agricultural University, Wuhan 430070, China; lijihong@ihb.ac.cn (J.L.); msldzh-6265@webmail.hzau.edu.cn (S.M.); bigfishyoo@webmail.hzau.edu.cn (W.Y.); zhoupeng92@webmail.hzau.edu.cn (P.Z.); 2020202040147@whu.edu.cn (X.Y.); yonganzhang@mail.hzau.edu.cn (Y.-A.Z.); 2 Institute of Hydrobiology, Chinese Academy of Sciences, Wuhan 430070, China; 3 College of Food Science and Technology, Huazhong Agricultural University, Wuhan 430070, China; mli_zhi@webmail.hzau.edu.cn (Z.L.); sharif@mail.hzau.edu.cn (M.S.I.); lijinquan@mail.hzau.edu.cn (J.L.); 4 College of Veterinary Medicine, Huazhong Agricultural University, Wuhan 430070, China; 5 Engineering Research Center of Green Development for Conventional Aquatic Biological Industry in the Yangtze River Economic Belt, Ministry of Education, Wuhan 430070, China

**Keywords:** *Aeromonas hydrophila*, live-attenuated vaccine, virulence, grass carp, immune response

## Abstract

*Aeromonas hydrophila* is an important pathogen that causes motile *Aeromonas* septicemia (MAS) in the aquaculture industry. Aerolysin, hemolysin, serine protease and enterotoxins are considered to be the major virulence factors of *A. hydrophila*. In this study, we constructed a five-gene (*aer*A, *hly*, *ahp*, *alt* and *ast*) deletion mutant strain (named *Aeromonas hydrophila* five-gene deletion strain, AHFGDS) to observe the biological characteristics and detect its potential as a live-attenuated vaccine candidate. AHFGDS displayed highly attenuated and showed increased susceptibility to fish blood and skin mucus killing, while the wild-type strain ZYAH72 was highly virulent. In zebrafish (*Danio rerio*), AHFGDS showed a 240-fold higher 50% lethal dose (LD50) than that of the wild-type strain. Immunization with AHFGDS by intracelomic injection or immersion routes both provided grass carp (*Ctenopharyngodon idella*) significant protection against the challenge of the strain ZYAH72 or J-1 and protected the fish organs from serious injury. Further agglutinating antibody titer test supported that AHFGDS could elicit a host-adaptive immune response. These results suggested the potential of AHFGDS to serve as a live-attenuated vaccine to control *A. hydrophila* infection in aquaculture.

## 1. Introduction

The ubiquitous bacterium *Aeromonas hydrophila* is an important Gram-negative, motile, rod-shaped bacterium belonging to the class of Gammaproteobacterias, order Aeromonadales and family Aeromonadaceae [1]. *A. hydrophila* can infect a wide variety of freshwater and marine fish, causing motile *Aeromonas* septicemia (MAS) [2]. The symptoms of MAS include swelling of tissues, dropsy, red sores, necrosis, ulceration and hemorrhagic septicemia [3]. MAS affects different fish species, including tilapia (*Oreochromis niloticus*), catfish (*Ictalrus punctatus*), goldfish (*Carassius auratus*), common carp (*Cyprinus carpio* L.) and eel (*Anguilla* spp.), which causes severe economic losses to the aquaculture industry through high mortality, weight loss and high treatment costs [4]. Grass carp (*Ctenopharyngodon idella*) is a fish species of the largest production in the world, accounting for ~16% of global freshwater aquaculture, and is an important economic species farmed extensively in China and other Asian countries. In recent years, MAS caused by *A. hydrophila* has become an increasingly prominent problem for the rapid development of grass carp industry [5].

To control MAS, antibiotic medicated feed is a general practice, which has been applicative in feeding infected fish [6]. However, many people questioned the use of antibiotics as a preventive measure because not only it could lead to the development of antibiotic resistance in many fish pathogens but also alter the intestinal flora [7].The transfer and emergence of drug resistance have occurred faster from aquatic bacteria to humans than from terrestrial animal bacteria to humans, which bring potential risk on human health [8]. Another important aspect is the fact that in 2019, the Ministry of Agriculture and Rural Affairs of the People’s Republic of China introduced a ban for antibiotic usage as growth promoters in animal production. Vaccine is an alternative and feasible control method to prevent MAS. The most extensively studied *A. hydrophila* vaccines are inactive vaccines. To note, the killed whole-cell vaccine against *A. hydrophila* (J-1 strain) obtained the national class I new veterinary drug certificate, making it the first aquatic bacterial vaccine in China. This vaccine obtained the veterinary drug production license from the Chinese government [(2011) 190986013] in 2011 [9]. Apart from inactive vaccines, recombinant protein vaccines such as *A. hydrophila* outer membrane proteins and bacterial lysates, DNA vaccines using carbon nanotubes or those that are yeast-based have been demonstrated to elicit protection against *A. hydrophila* challenge [10,11,12,13]. Despite the fact that vaccination represents the most effective strategy to prevent diseases in the aquaculture industry, commercial vaccines for *A. hydrophila* in fish still have been a challenge because of its biochemical and serological heterogeneity [14].

Live bacterial vaccines have the advantages of their mimicry of a natural infection, intrinsic adjuvant properties and their possibility to be administered via needle-free delivery systems [15]. To develop effective live bacterial vaccines, targeted mutagenesis of virulence genes strategy has been successfully used in various bacterial species. Considering that *A. hydrophila* pathogenicity is closely related to surface properties and extracellular enzymes [16,17], we selected five genes including aerolysin (*aer*A), hemolysin (*hly*), serine protease (*ahp*), heat-labile cytotonic enterotoxin (*alt*) and cytotonic enterotoxin (*ast*) to construct a live vaccine strain. The coding products of these five genes are involved in virulence of the pathogen [18]. According to a previous study, a higher frequency of genetic profile *aer*A^+^*alt*^+^*ahp*^+^ was determined in the isolates from diseased animals compared to those from healthy fish or water environments, and *aerA*^+^*alt*^+^*ahp*^+^ isolates exhibited higher virulence in zebrafish [19]. Additionally, hemolysins are a diverse group of multifunctional enzymes that play a central role in *A. hydrophila* pathogenesis. Aerolysin A (*aer*A) and hemolysin A (*hly*A) comprise a two-component hemolytic system in which virulence is attenuated only when both *hly*A and *aer*A activity is abolished [20].

In this study, a five-gene (*aer*A, *alt*, *ahp*, *ast* and *hly*) deletion mutant strain (named *Aeromonas hydrophila* five-gene deletion strain, AHFGDS) was constructed based on the highly virulent strain ZYAH72. AHFGDS showed no growth defect, but totally lost hemolytic capacity and displayed significantly lower cytotoxicity. The safety of AHFGDS was determined both in vitro and in vivo. We further evaluated the immunoprotective potential of this strain. The results showed that when immunized either by immersion or by injection route, AHFGDS can induce high levels of protection against *A. hydrophila* infection in grass carp. This study illuminates the feasibility of multigene deletion strategies in the development of vaccines against *A. hydrophila* infection.

## 2. Materials and Methods

### 2.1. Declaration of Ethical Approval

All animal experimental procedures were strictly carried out according to the recommendations in the Guide for the Care and Use of Laboratory Animals of Hubei Province, China. The animal experiment protocol (HZAUFI-2019-023) was approved by the Laboratory Animal Monitoring Committee of Huazhong Agricultural University. All efforts were made to minimize animal distress.

Healthy grass carp (12 months of age, with an average weight of ~70 g) purchased from Xiantao Hatchery (Hubei, Wuhan, China) were maintained and acclimated to recirculating tanks (1000 L, 28 ± 1 °C) containing filtered and oxygenated water for at least two weeks before experiments. Each fish was visually inspected externally to make sure it was clinically healthy according to the United States Environmental Protection Agency (EPA) guidelines for qualitatively assessing fish health [21]. Grass carp were fed daily with commercially produced food pellets (Haida, China) under natural photoperiod. Water temperature was maintained at 23–25 °C. AB line wild-type zebrafish used in this work were from the Institute of Hydrobiology, Chinese Academy of Sciences (Wuhan, China). Zebrafish were maintained at a density of 10 fish/tank in 8 L tanks. They were fed with commercial feed for aquatic animal twice per day under natural photoperiod. The water temperature was maintained at 24–26 °C during cultivation. The fish were acclimatized with freshwater for two weeks before experiments was performed.

### 2.2. Bacterial Strains and Growth Conditions

The strains and plasmids used in this study were listed in Table 1. *A. hydrophila* ZYAH72 was isolated from diseased crucian carp in Hubei province, China in 2014. ZYAH72 (NCBI accession No: NZ_CP016989) and its mutant strain were cultured in Luria-Bertani (LB) broth or on LB agar (LA) plates at 28 °C, supplemented with chloramphenicol (50 mg/mL) (Solarbio, #722F041) and 7% sucrose (SCR, #10021418) when required. *Escherichia* coli χ7213 strain was cultured in LB broth at 37 °C, supplemented with diaminopimelic acid (50 mg/mL) (Sigma, #33240).

### 2.3. Construction of the A. hydrophila Five-Gene Deletion Mutant and Phenotype Characterization

Five target genes were deleted by an allelic replacement strategy. The target genes and primers used in this study are listed in Table S1. The primers were designed referring to the complete genome sequence of *Aeromonas hydrophila* strain ZYAH72. Upstream and downstream flanking fragments of *ast* were amplified by PCR using primers P1/P2 and P3/P4, respectively. The fusion of the two fragments was amplified by overlap PCR using primers P1/P4. The overlap fragment was ligated into pRE112 at the *Xba*I/*Sac*I sites and then sequenced. The resulting plasmid pRE112-ast was transformed into *E. coli* χ7213 for mobilization into *A. hydrophila* via conjugation. The transconjugants containing plasmid pRE112-ast integrated into ZYAH72 strain chromosome by a single crossover event were selected on LA media containing chloramphenicol. Allelic exchange between the chromosomal gene and the mutagenized plasmids copy was achieved by the second crossover event and was counter-selected on LB containing 7% sucrose to determine the excision of pRE112 from the chromosome. By chloramphenicol sensitivity and sucrose resistance, ∆*ast* mutant was selected. Mutant was verified by PCR using primers P5/6 and P7/8 and direct DNA sequencing of the mutation sites. Additional deletions of *alt*, *ahp*, *hly* and *aer*A were performed sequentially to generate the five-gene deletion mutant.

To determine the growth kinetics of different strains, 1:100 diluted overnight cultures were cultured in LB medium at 28 °C. Samples were taken every half hour, and the optical densities were measured at 600 nm (OD_600 nm_). The experiment was performed three times. β-hemolytic phenotype of different strains was confirmed on sheep’s blood agar (HKM, #CP0800). Cytotoxicity was measured using LDH Cytotoxicity Assay Kit (Promega, #0000332826). Grass carp kidney cell line (CIK) cell monolayers were washed twice with 28 °C preheated PBS and infected at a multiplicity of infection (MOI) of 5 in 96-well tissue culture plates, respectively (Corning Inc., Corning, NY, USA). CIK cells were incubated with the ZYAH72 and AHFGDS at a 5 MOI for 2 h, and supernatants were collected for measuring the LDH release. The percentage of cytotoxicity was calculated as recommended by the manufacturer using the following formula: [(OD_490 nm_ sample − OD_490 nm_ spontaneous)/(OD_490 nm_ maximum release − OD_490 nm_ spontaneous)] × 100. OD_490 nm_ spontaneous indicated LDH release from uninfected cells into the culture supernatant and maximum release denoted LDH release obtained by lysis of the uninfected cells. Three independent experiments were performed in duplicate wells.

### 2.4. Blood and Skin Mucus Killing Assay

Blood killing assay was carried out according to the procedures described previously [24]. The experiments were performed in triplicate. Blood of grass carp was exsanguinated with a sterile syringe and heparinized immediately. Heparinized blood (900 μL) was combined with 100 μL exponential phase *A. hydrophila* strain cultures at a concentration of 5 × 10^4^ CFU/mL. The mixtures were incubated at 28 °C. Samples (100 μL) were taken every half hour and diluted in normal saline (900 μL) serially. The suspensions were plated on LA agar and incubated overnight at 28 °C. Colonies were counted after 24 h.

The mucus sample was prepared as previously described [25]. Mucus was taken from ten zebrafish at regular intervals. Skin mucus was carefully scraped with a rubber spatula, thoroughly mixed with equal quantity of sterilized PBS and centrifuged at 20,000× *g* for 30 min twice at 4 °C. The supernatant was filtered with a 0.22 μm sterile filter. Three hundred microliters mucus was mixed with 300 μL *A. hydrophila* at a final concentration of 1.5 × 10^5^ CFU/mL. The mixtures were incubated at 28 °C. Samples (100 μL) were taken after 1 h and diluted in normal saline (900 μL) serially. The suspensions were plated on LA agar and incubated overnight at 28 °C. Colonies were counted after 24 h. The experiments were performed in triplicate.

### 2.5. Median Lethal Dose (LD50) Assays

To examine the lethal dose 50% (LD_50_) of AHFGDS and wild-type strain ZYAH72, zebrafish were divided randomly into 10 groups (10 fish/group), and each group was injected by the intracelomic (i.c.) route with 3.4 × 10^5^, 6.8 × 10^4^, 1.4 × 10^4^, 2.7 × 10^3^, 5.4 × 10^2^ CFU/fish of wild-type strain ZYAH72 and 4.3 × 10^7^, 8.6 × 10^6^, 1.7 × 10^6^, 3.4 × 10^5^, 6.8 × 10^4^ CFU/fish of AHFGDS, respectively. Five zebrafish injected with PBS were set as the injection control group. The fish were observed for 14 days to determine the survival rate. Surviving fish were sacrificed on day 14 post-infection. LD_50_ values were calculated according to Karber’s methods. To minimize the use of animal, LD_50_ assay was done in duplicate.

### 2.6. Quantitative Real-Time Reverse Transcriptase-PCR (qRT-PCR)

Grass carp were divided into AHFGDS infection group, wild-type ZYAH72 infection group and PBS control group, three fish per group. The infection dose was 2.0 × 10^6^ CFU/fish by i.c. route. The kidney, head kidney and spleen were collected 12 hpi (hours post infection), and every organ sample was divided into three pieces. The samples were extracted the total RNA immediately. Total RNA was extracted by Trizol (Invitrogen, #15596-026) according to the instructions of manufacturer. A reverse transcription kit (Vazyme, #R323-01) was used to eliminate genomic DNA contaminant and to obtain cDNA. The transcription level of inflammation-associated cytokines (TGF-β, IL-1β and TNF-α) were assessed by real-time quantitative PCR. RT-qPCR was performed by SYBR green real-time PCR mix (Bio-Rad, #170–8894) using a CFX real-time PCR detection system (Bio-Rad, Hercules, CA, USA) following the manufacturer’s instructions. PCR conditions were as follows: 95 °C for 5 min and then 45 cycles of 95 °C for 20 s, 60 °C for 20 s and 72 °C for 20 s. Primers used for each gene are listed in the Supplementary Table. The relative expression of each immune-relative gene was determined by comparing to the expression level of β-actin using the ΔΔCt method, samples were analyzed in triplicate, and all data were reported as relative mRNA expression compared to the value of the PBS control. The experiments were performed three times independently.

### 2.7. Vaccination and Challenge Assay

*A. hydrophila* strains were cultured in LB medium to 0.8 of OD_600 nm_, washed with PBS, and then resuspended in PBS to proper concentration. The grass carp were divided randomly into three groups (40 fish/group). The first group was immunized with 3 × 10^8^ CFU/mL of AHFGDS via immersion (imm.) route for 10 min. The second group immunized with 2 × 10^7^ CFU/fish of AHFGDS via i.c. route. The third group was set as control group. At week 3, the fish were boosted with the same dose and immunization routes of AHFGDS. At two weeks post-booster immunization, blood samples were collected, randomly selected, from five fish per group by caudal vein puncture. Serum was carefully collected after a centrifugation at 10,000× *g* for 10 min for later agglutinating antibody titer test. Forty fish per group were divided randomly into two groups, respectively, and challenged with 2 × 10^7^ CFU/fish ZYAH72 or 2 × 10^8^ CFU/fish J-1 via i.c. route. Percent survival was recorded daily up to 14 d and calculated percent survival. The experiments were performed in triplicate. The relative percent survival (%, RPS) was calculated as follows: (1−percent mortality in the experimental group/percent mortality in the control group) × 100.

### 2.8. Agglutinating Antibody Titer

The agglutinating titers of serum antibody were determined according to the previous study [26]. Firstly, 50 µL serum was serially diluted with 0.85% NaCl in a 96-well microtiter plate with thorough mixing. Then, 50 µL AHFGDS (10^9^ CFU/mL) was mixed into each well, and the whole plate was incubated at 28 °C for 2 h and then stored at 4 °C overnight. The agglutination reaction was observed at 20× magnification under a dissecting microscope, and the last dilution of serum giving a visible precipitation was taken as the agglutinating antibody titer.

### 2.9. Histopathological Studies

Histopathological sections were prepared to investigate whether AHFGDS immunization could reduce tissue injury caused by *A. hydrophila* infection. Six grass carp were divided into an AHFGDS injection-immunized group and control group (3 fish/group). The immunization and challenge process was as described in 2.7. Intestine, spleen, head kidney and trunk kidney samples were collected 48 h after infection and were fixed in 10% formalin (buffer PBS; pH 7.2) for 24 h. Following fixation, the samples were dehydrated with ethanol, cleared with xylene and infiltrated with paraffin. After paraffin embedding, blocks were processed to obtain 4 µm sections, which were stained with a standard hematoxylin and eosin method. Stained samples were examined by light microscopy (Nikon, Japan). We analyzed tissues from three fishes per group for histopathology, and data from representative tissues are presented.

### 2.10. Statistical Analysis

Statistical analysis was performed by GraphPad Prism 6 (Graph Pad Software, Inc, San Diego, CA, USA). Survival data were analyzed with the log-rank (Mantel–Cox) test. The statistical p values were calculated by the two-tailed Mann–Whitney t test. Differences were considered significant at *p* < 0.05 and highly significant at *p* < 0.01.

## 3. Results

### 3.1. Construction and Characterization of the A. hydrophila Mutant AHFGDS

**Figure 1:**
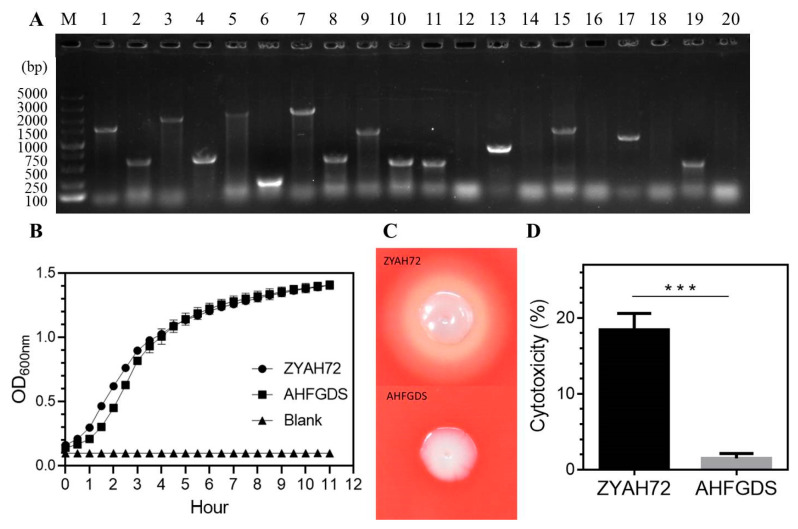
Phenotype characterization of AHFGDS. (**A**) Confirmation of strain AHFGDS by PCR using detecting primers covering (lane 1–10) or within (lane 11–20) the in-frame deletion fragments of the genes *ast*, *alt*, *ahp*, *hly* and *aer*A. Lane 1, 3, 5, 7, 9, 11, 13, 15, 17, 19, ZYAH72 with primer sets P5/P6, P13/P14, P21/P22, P29/P30, P37/P38, P7/P8, P15/P16, P23/P24, P31/P32, P39/P40, respectively. Lane 2, 4, 6, 8, 10, 12, 14, 16, 18, 20, AHFGDS with above primer sets, respectively. Marker is shown to the left (M). (**B**) Growth curves in LB medium over 12 h period. (**C**) β-hemolytic phenotype of the strains grown on sheep’s blood agar. (**D**) Cytotoxicity results after 2 h of incubation with the CIK cell line. The results were presented as the mean ± SD. *** *p* < 0.001.

To generate a fully attenuated live vaccine strain, we constructed the mutant with deletion in the region covering the genes *ast*, *alt*, *ahp*, *hly* and *aer*A that play important roles in the bacterial pathogenesis. The PCR identification (Figure 1A) and direct DNA sequencing showed that the mutant strain was successfully constructed and the subsequent mutant strain (Δ*ast*Δ*alt*Δ*ahp*Δ*hly*Δ*aer*A) renamed as AHFGDS. We compared the growth of AHFGDS with ZYAH72 under normal culture conditions, and the mutant strain exhibited no growth defects (Figure 1B). However, the AHFGDS had reduced almost all hemolytic activities (Figure 1C) and also failed to show cytotoxicity to CIK cell line (1.47%) comparing to ZYAH72 (18.42%) (Figure 1D). Taken together, these results showed that the deletion of five genes had no negative impact on growth but significantly reduced the bacterial hemolytic activities and cytotoxicity, implicating attenuated virulence of AHFGDS.

### 3.2. AHFGDS Was Sensitive against Host Clearance

**Figure 2:**
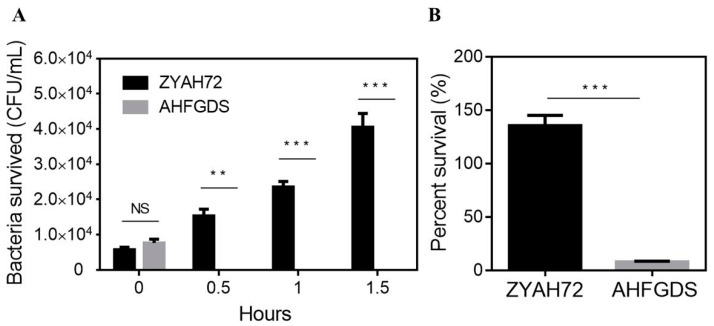
Blood (**A**) and skin mucus (**B**) killing assay of wild-type ZYAH72 and AHFGDS mutant. The experiments were performed three times independently. The data shown were obtained from a representative experiment and presented as the mean ± SD. NS: not significant, ** *p* < 0.01, *** *p* < 0.001.

To evaluate the resistance against host immune clearance of AHFGDS, fish blood and skin mucus killing assays were applied. After incubation with heparinized grass carp blood, the number of AHFGDS was reduced by 99.7% after 0.5 h, and no alive bacteria were detected after 1.5 h, while wild-type ZYAH72 grew by 7.11-fold until the end of the assay (Figure 2A). Similarly, AHFGDS showed significantly reduced resistance against zebrafish mucus killing comparing to wild-type ZYAH72. Only 7.9% bacteria of AHFGDS survived after one hour incubation with fish mucus, while wild-type ZYAH72 increased to 135.5% (Figure 2B).

### 3.3. AHFGDS Was Highly Attenuated in Zebrafish

**Figure 3:**
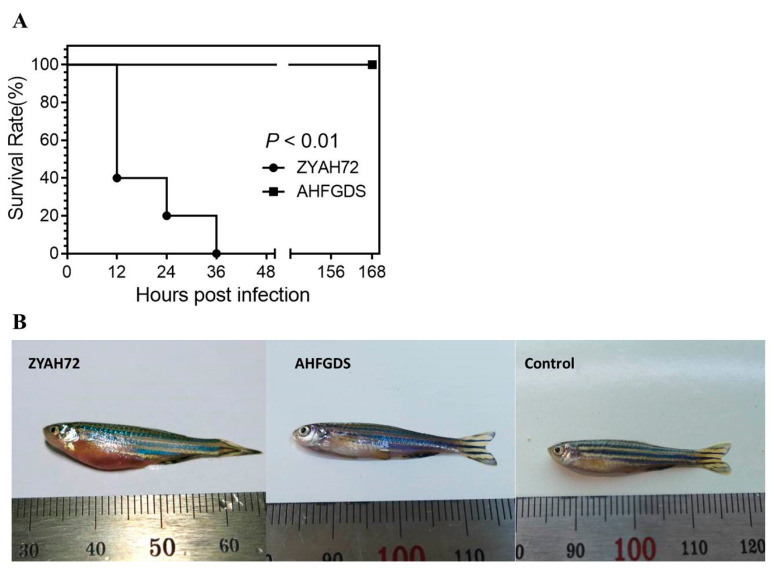
(**A**) Survival curves for zebrafish challenged with wild-type ZYAH72 and AHFGDS at doses of 3.4 × 10^5^ CFU/fish. (**B**) Symptoms of zebrafish infected by wild-type ZYAH72, AHFGDS or control group.

**Figure 4:**
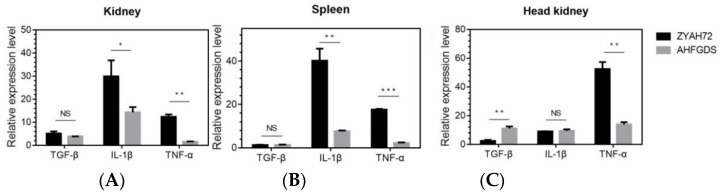
The mRNA levels of TGF-β, IL-1β and TNF-α coding genes in grass carp trunk kidney (**A**), spleen (**B**) and head kidney (**C**) of AHFGDS or wild-type ZYAH72 infected group. The mRNA level of each gene was normalized to that of β-actin. The experiments were performed three times independently. The data shown were obtained from a representative experiment. NS: not significant, * *p* < 0.05, ** *p* < 0.01, *** *p* < 0.001.

Previous results indicated the attenuation of AHFGDS, we further explored its virulence in zebrafish. The LD_50_ value of AHFGDS was 2.8 × 10^6^ CFU/fish, which was 240-fold higher than that of wild-type ZYAH72; therefore, the virulence of AHFGDS was proved to be significantly reduced (Table 2). The fish in the control group all survived until the end of the study. At doses of 3.4 × 10^5^ CFU/fish, the wild-type ZYAH72 infection resulted in a rapid onset of the disease and high fatality, all fish died during the first 36 h, while all fish from AHFGDS group survived (Figure 3A). The fish of ZYAH72 group exhibited typical symptoms of hemorrhagic septicemia and distended abdomen, but the AHFGDS group exhibited no obvious symptoms (Figure 4B). Taken together, these results suggested the significantly attenuated virulence and high-level safety of AHFGDS in vivo.

### 3.4. AHFGDS Elicited Weakened Inflammatory Responses

Excessive inflammatory cytokine production can lead to tissue damage, organ failure and ultimately death. To test the inflammatory responses AHFGDS elicited, we assessed the transcription level of inflammation-associated cytokines (TGF-β, IL-1β and TNF-α) in grass carp infected with AHFGDS or wild-type ZYAH72 (Figure 4). In kidney and spleen, comparing to ZYAH72 group, the transcripts of pro-inflammatory cytokines IL-1β and TNF-α were all significantly reduced in AHFGDS group, while the transcripts of TGF-β displayed no difference. In the immune organ head kidney, the transcripts of anti-inflammatory cytokine TGF-β induced by AHFGDS infection increased 4.7-fold (*p* < 0.01) comparing to ZYAH72 infection group, while pro-inflammatory cytokines TNF-α level decreased 3.8-fold (*p* < 0.01).

### 3.5. AHFGDS Offered Grass Carp Effective Protection against A. hydrophila Infection

**Figure 5:**
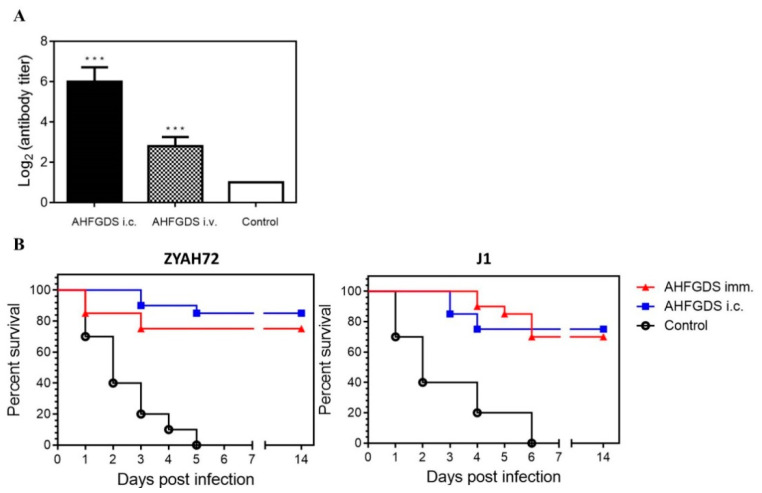
(**A**) Serum agglutinating antibody titers in grass carp immunized with AHFGDS through different administration routes. (**B**) Immunization with AHFGDS protects grass carp against lethal challenges with wild-type ZYAH72 or J-1. *** *p* < 0.001.

The delicate balance between attenuation and immunogenicity is critical for the success of an attenuated live vaccine. Considering AHFGDS showed significantly reduced virulence, we further explored its immunogenicity. The specific immune response in grass carp after vaccination was evaluated by measuring serum agglutinating antibody titer. All the immunized fish exhibited significantly higher agglutinating antibody titers in comparison with the control (Figure 5A). Fish immunized via i.c. route displayed higher antibody level (6.0 ± 0.70) than imm. route (2.8 ± 0.45). We further evaluated the immune protection mediated by AHFGDS in grass carp via imm. or i.c. route. At 2 weeks post-booster immunization, fish were challenged i.c. with a lethal dose of wild-type ZYAH72 (2 × 10^7^ CFU/fish) or J-1 (2 × 10^8^ CFU/fish). The relative percent survivals (RPSs) of grass carp vaccinated via AHFGDS immunization bath were 75 and 70% against ZYAH72 or J-1 challenge, respectively (Figure 5B). Similarly, the protection rates of AHFGDS via i.c. immunization route, in terms of RPS were 85 and 75% against ZYAH72 or J-1 infection, respectively (Figure 5B). These data showed that AHFGDS vaccinated fish were effectively protected via either i.c. or imm. route.

**Figure 6:**
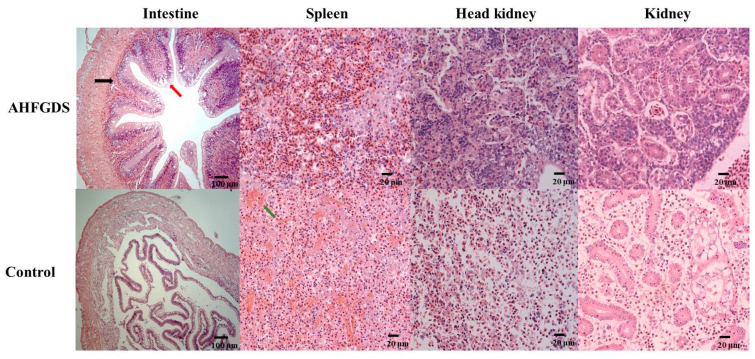
Histopathological examinations. Samples collected from fish vaccinated with AHFGDS or PBS (control group) at 48 h post wild-type ZYHA72 infection. The tissues were stained with hematoxylin and eosin. Black arrow indicates lymphocyte recruitment, red arrow indicates goblet cells, and green arrow indicates red blood cell necrosis.

Histopathological sections were prepared to investigate whether AHFGDS immunization could reduce tissue injury caused by *A. hydrophila* infection. Intestine, spleen, head kidney and trunk kidney samples were collected 48 h after grass carp were challenged i.c. with lethal dose of wild-type ZYAH72 (Figure 6). In intestine, the control group showed denudation of the epithelium and collapse of villous structure, while the intestine tissue of AHFGDS immune group displayed normal structure with increased goblet cells. In spleen and head kidney, the control group showed tissue collapse and extravasation of red blood cell, while AHFGDS immune group showed normal form. In kidney, tubular and tubules necrosis were observed in the control group, while the AHFGDS immune group only exhibited slight detachment of renal tubules from the basement membrane. Taken together, AHFGDS immunization could effectively protect fish issues from injury caused by *A. hydrophila* infection.

## 4. Discussion

With the increasing immunological understanding and development of molecular techniques, live vaccines have gained renewed interest within the last 20 years. Live vaccines have the advantages of mimicking the route of pathogen infection and stimulating the mucosal immune response, and also enriching the administration routes [15]. In most of these cases, specific genes that are essential to central metabolism and pathogenicity were selected to be mutated to attenuate the pathogen virulence. Previous studies supported that pathogenicity of *Aeromonas* ssp. is multifactorial, involving different genes products acting individually or together [27]. In this study, we constructed an *A. hydrophila* mutant AHFGDS with deletion in the regions covering the genes *aer*A, *hly*, *ahp*, *alt* and *ast*. Aerolysin, hemolysin, serine protease and enterotoxins, the products of the above selected encoding genes, have been revealed as the major virulence factors and are important for the pathogenesis of *A. hydrophila*. The distribution of two hemolytic toxins (aerolysin and hemolysin) was first reported from clinical and environmental *Aeromonas* spp. isolates [28]. Absent in the *A. caviae* and *A. veronii* groups, aerolysin was found to be present in 91% of *A. hydrophila* isolates [29]. Wong et al. suggested that the hemolytic activity of *A. hydrophila* is related to both the hemolysin and the aerolysin genes, and only the *hly*A *aer*A double mutant showed a statistically significant reduction in virulence, with a 20-fold change in LD_50_ [20]. Apart from hemolytic cytotoxins, cytotonic enterotoxins also contribute to the pathogenesis of *Aeromonas* ssp. Sha et al. demonstrated that heat-labile (*alt*) and the heat-stable (*ast*) cytotonic enterotoxin were responsible for *A. hydrophila*-induced gastroenteritis in mice [30]. A previous study revealed that *aer*A^+^*alt*^+^*ahp*^+^ was a more frequent virulence genotype in *A. hydrophila* isolates from clinical diseases than from a healthy fish and water environment, and the *aer*A^+^*alt*^+^*ahp*^+^ isolates were the more virulent to zebrafish compared to the other six genetic profiles [19]. The encoding product of *ahp* is a serine protease, which has been reported to activate the aerolysin and other extracellular enzymes, thus affecting the overall virulence of aeromonads [31].

Safety is one of main issues in the development of live-attenuated vaccines [32]. An ideal vaccine does not cause disease or negative side effects in the host and, at the same time, can induce an effective immune response that is capable of protecting against the pathogen. To evaluate the pathogenicity of AHFGDS, we performed a series of in vitro and in vivo assays. In the first place, we ruled out the possible impact of gene deletion on growth. The AHFGDS mutant exhibited no difference in growth compared with wild-type ZYAH72, indicating that these five genes were not required for nutrient acquisition under culture conditions. However, AHFGDS showed drastically decreased hemolytic activities and cytotoxicity and significantly reduced the ability to escape killing by host immune system. In zebrafish infection model, the LD_50_ value of AHFGDS was 240-fold higher than that of ZYAH72, indicating significantly reduced pathogenicity of AHFGDS. Many strategies had been pursued by researchers to construct a live-attenuated *A. hydrophila* vaccine strain. Strain XX1LA was generated as a live-attenuated vaccine candidate by rifampicin passage of pathogenic *A. hydrophila* strain XX1. The LD_50_ showed a 200-fold virulence reduction [33]. In another study, the transposon insertion mutagenesis approach was applied to obtain an exoenzyme mutant of *A. hydrophila* strain J-1. The mutant strain was deficient in protease, hemolysin, amylase and DNase. However, the degree of virulence reduction was not mentioned [34]. Swain et al. passaged continuously over a period of 8 years to get two smooth virulent types of *A. hydrophila*. The LD_50_ was 10^5.5^ and 10^6^ CFU/mL^−1^ for parent smooth type *A. hydrophila*, respectively, whereas it was 10^11^ and 10^11.5^ CFU/mL^−1^ for *A. hydrophila* rough type, respectively [35]. The major safety risk of live vaccines is the theoretically possible reversion to their original pathogenic (disease-causing) form. Differently from previous studies, AHFGDS was constructed via homologous recombination strategy and defined principal virulence genes (*aer*A, *alt*, *ahp*, *ast* and *hly*) were in-frame deleted, which minimized the reverse risk to the utmost extent. To ensure the safety, further genetic modification could be applied on an AHFGDS strain.

We further explored the reduced virulence of AHFGDS from perspective of the host innate immune response. Comparing to wide-type ZYAH72 infection group, the mRNA levels of pro-inflammatory cytokine tumor necrosis factor-α (TNF-α) and interleukin-1β (IL-1β) in the kidney and spleen of AHFGDS infection group were significantly reduced. Additionally, in head kidney, AHFGDS induced moderate transcription of anti-inflammatory cytokine transforming growth factor-β (TGF-β). On one hand, excessive inflammatory cytokine production can lead to tissue damage, hemodynamic changes, organ failure and ultimately death [27]. However, controllable inflammation evoked by AHFGDS could effectively contribute to bacterial clearance, which explains the reduced virulence. The reduced inflammation response could be due to the lack of hemolysin, aerolysin and enterotoxins, which are regarded as the major causes of inflammation [36]. On the other hand, innate immune response elicited by a live-attenuated vaccine could modulate the quantity and quality of long-term T and B cell memory and protective immune responses to pathogens [37]. IL-1β and TNF-α, as typical pro-inflammatory cytokines, can propel the growth and proliferation of immune cells [38]. TGF-β is a key regulator of host defense that performs an essential role in immune function modulation in both the innate and adaptive immune pathway [39]. To sum up, AHFGDS showed good characteristics to activate host immune response without causing excessive inflammatory response leading to tissue damage.

In the aquatic industry, vaccines are administered either orally, by immersion or by injection route [40]. Injection vaccinations have the advantages of low dose usage of antigen, effective vaccination rate and enhanced immunogenicity of the vaccine via the addition of an adjuvant [9]. However, limitations also exist, including stress to fish during vaccination, expensive labor cost, specification requirements for fish, antigenic competition and interference between antigens and unknown side effects [9]. Vaccination via the mucosal delivery routes can ease the administration of antigens and is less stressful than the injection delivery routes [41]. The injection and immersion immunoprotection efficacy of AHFGDS was further evaluated in grass carp. The results showed, either via injection or immersion, that AHFGDS could elicit host-adaptive immune response. In the first week of immunization, the antibody titer began to rise and remained high until the end of the experiment (data not shown), indicating AHFGDS vaccine candidate could induce specific immunity responses. To note, the injection immunized group exhibited significantly higher agglutinating antibody titers in comparison to the immersion group, which is consistent with a previous study on *Vibrio alginolyticus* [26]. Compared to the non-vaccinated group, fish vaccinated via both routes were all effectively protected with RPS over 70% after challenged with prevalent strains ZYAH72 or J-1, while all unvaccinated fish died upon the bacterial challenge. Considering the antibody titer induced by immersion vaccination was only half of that induced by injection, the protection efficacy conducted via immersion was also quite impressive. During immersion vaccination, the antigens are taken up by the skin, gills or gut and processed by the immune system, where the resulting response may lead to protection [42]. Shoemaker et al. indicated that a simple formalin-killed vaccine administered via immersion exposure provided significant protection in juvenile hybrid catfish against vAh, supporting the feasibility of immersion immunization route against *A. hydrophila* infection [43]. After immersion vaccination, there is a so-called disparity when it comes to antibody responses: mucosal immunization may induce localized mucosal immune responses [42], which may explain the differential performance of agglutinating antibody titers in grass carp blood. In further study, another administration route such as oral route could also be tested for AHFGDS. Additionally, the combination of a natural feed component seems a promising way to enhance the protection effect, as they show good potential in bacteria disease prevention [44,45].

To verify the protection effect of AHFGDS on tissue damage induced by *A. hydrophila* infection, histological analysis was carried out. *A. hydrophila* is generally considered a major pathogen causing intestinal inflammation in fish, which leads to bacterial enteritis, the most common intestinal disease suffered by freshwater fish [46]. In intestine, we observed epithelial cell layer with increased goblet cells but no severe inflammation in AHFGDS immunized group, while the control group showed a collapse of villous structure, which was consistent with typical clinical *A. hydrophila* infection phenotype. In kidney, spleen and head kidney, the control group displayed varying degrees of necrosis and hemorrhaging, which is also the typical symptom of mobile *Aeromonas* septicemia, while AHFGDS group was well protected. It can, therefore, be concluded that AHFGDS provided a safe and reliable way that can effectively improve the specific immunity ability of grass carp.

## 5. Conclusions

In conclusion, we successfully constructed a multiple gene in-frame deletion *A. hydrophila* strain AHFGDS, then proved AHFGDS displayed decreased hemolytic activities and cytotoxicity without affecting growth rate. AHFGDS showed higher susceptibility to host immune clearance and was significantly attenuated in a zebrafish infection model. To assess the potential as a live vaccine, AHFGDS was able to elicit a host-adaptive immune response and provide effective protection against *A. hydrophila* infection in grass carp via different immunization routes. These results indicated that AHFGDS has the potential to be developed as a live vaccine candidate in the control of MAS caused by *A. hydrophila* in a more labor- and cost-efficient way in the aquaculture industry.

## Figures and Tables

**Table 1:** Strains and plasmids used in this study.

Strains and Plasmids	Description	Source
Strains		
*Aeromonas hydrophila*		
ZYAH72	Wild type	Lab collection
AHFGDS	ZYAH72 derivative, Δ*ast*Δ*alt*Δ*aph*Δ*hly*Δ*aer*A	This work
*Escherichia coli*		
χ7213	*thr*-1 *leu*B6 *fhu*A21 *lac*Y1 *gln*V44 *rec*A1 Δ*asd*A4 Δ(*zhf*-2::Tn10) *thi*-1	[22]
Plasmids		
pRE112	Suicide vector, *sac*B, mob-(RP4)R6K ori, Cmr	[23]
pRE112-ast	pRE112 derivative, designed for knockout of *ast*, Cmr	This work
pRE112-alt	pRE112 derivative, designed for knockout of *alt*, Cmr	This work
pRE112-aph	pRE112 derivative, designed for knockout of *aph*, Cmr	This work
pRE112-hly	pRE112 derivative, designed for knockout of *hly*, Cmr	This work
pRE112-aerA	pRE112 derivative, designed for knockout of *aer*A, Cmr	This work

**Table 2:** Calculations of LD50s of the ZYAH72 and AHFGDS strains in zebrafish.

Dose of Challenge CFU	Number of Death/Total		Survival Rate (%)	
	ZYAH72	AHFGDS	ZYAH72	AHFGDS
4.3 × 10^7^	-	10/10	-	0
8.6 × 10^6^	-	8/10	-	20
1.7 × 10^6^	-	4/10	-	60
3.4 × 10^5^	10/10	0/10	0	100
6.8 × 10^4^	10/10	0/10	0	100
1.4 × 10^4^	4/10	-	60	-
2.7 × 10^3^	2/10	-	80	-
5.4 × 10^2^	0/10	-	100	-
LD_50_ *	1.2 × 10^4^	2.8 × 10^6^		

* The LD_50_ was calculated according to Karber’s method.

## Supplementary Materials

The following are available online at https://www.mdpi.com/article/10.3390/vaccines9050451/s1. Table S1: Primers used in this study.
